# Which bronchodilator reversibility criteria can predict severe acute exacerbation in chronic obstructive pulmonary disease patients?

**DOI:** 10.1186/s12931-017-0587-9

**Published:** 2017-05-30

**Authors:** Junghyun Kim, Woo Jin Kim, Chang-Hoon Lee, Sang Haak Lee, Myung-Goo Lee, Kyeong-Cheol Shin, Kwang Ha Yoo, Ji-Hyun Lee, Seong Yong Lim, Ju Ock Na, Hun-Gyu Hwang, Yoonki Hong, Myoung Nam Lim, Chul-Gyu Yoo, Ki Suck Jung, Sang-Do Lee

**Affiliations:** 1 Division of Pulmonary and Critical Care Medicine, Department of Internal Medicine, National Medical Center, Seoul, Republic of Korea; 2 Department of Internal Medicine and Environmental Health Center, Kangwon National University Hospital, Chuncheon, Republic of Korea; 3 Division of Pulmonary and Critical Care Medicine, Department of Internal Medicine, Seoul National University Hospital, 101 Daehak-Ro, Jongno-Gu, Seoul, 03080 Republic of Korea; 4 Department of Internal Medicine, The Catholic University of Korea, St. Paul’s Hospital, Seoul, Republic of Korea; 5 Division of Pulmonary, Allergy & Critical Care Medicine, Hallym University Chuncheon Sacred Heart Hospital, Chuncheon, Republic of Korea; 6 Division of Pulmonology and Allergy, Regional Center for Respiratory Disease, Yeungnam University Medical Center, Daegu, Republic of Korea; 7 Division of Pulmonary and Critical Care Medicine, Department of Internal Medicine, Konkuk University Medical Center, Konkuk University School of Medicine, Seoul, Korea; 8 Division of Respiratory and Critical Care Medicine, Department of Internal Medicine, CHA Bundang Medical Center, CHA University, Seongnam, Republic of Korea; 9 Division of Pulmonary and Critical Care Medicine, Department of Medicine, Kangbuk Samsung Hospital, Sungkyunkwan University School of Medicine, Seoul, Republic of Korea; 10 Department of Pulmonary Medicine, Soonchunhyang University Cheonan Hospital, Cheonan-si, Republic of Korea; 11 Department of Medicine, Soonchunhyang University Gumi’s Hospital, Gumi, North Kyungsang Province Republic of Korea; 12 Division of Pulmonary Medicine, Department of Internal Medicine, Hallym University Sacred Heart Hospital, Hallym University Medical School, Anyang, Republic of Korea; 13 Division of Pulmonary and Critical Care Medicine, Department of Internal Medicine, Asan Medical Center, University of Ulsan College of Medicine, Seoul, Republic of Korea

**Keywords:** Bronchodilator reversibility, COPD, Severe acute exacerbation

## Abstract

**Background:**

It is unclear whether various bronchodilator reversibility (BDR) criteria affect the prognosis of chronic obstructive pulmonary disease (COPD). The aim of this study is to evaluate the impact of positive BDR defined according to various BDR criteria on the risk of severe acute exacerbation (AE) in COPD patients.

**Methods:**

Patients from four prospective COPD cohorts in South Korea who underwent follow-up for at least 1 year were enrolled in this study. The assessed BDR criteria included the Global Initiative for Chronic Obstructive Lung Disease (GOLD), American Thoracic Society (ATS), American College of Chest Physicians, (ACCP), major criteria of the Spanish definition of asthma-COPD overlap syndrome (ACOS), criteria compatible with ACOS in the Global Initiative for Asthma (GINA), and European Respiratory Society (ERS). The rate of patients with severe AE who required hospitalization within 1 year due to BDR results according to each set of criteria was analyzed using logistic regression models.

**Results:**

Among a total of 854 patients, the BDR-positive cases varied according to the criteria used. There was a 3.5% positive BDR rate according to GINA and a 29.9% rate according to the ATS criteria. Positive BDR according to the GOLD criteria was significantly associated with a decreased risk of severe AE (adjusted odds ratio (aOR) = 0.38; 95% Confidence interval (CI) = 0.15–0.93). This result remained statistically significant even in a sensitivity analysis that included only participants with a smoking history of at least 10 pack-years and in the analysis for the propensity score-matched participants.

**Conclusions:**

Among different criteria for positive BDR, the use of the GOLD ones was significantly associated with a decreased risk of severe AE in COPD patients. Increase use of ICS/LABA may have affected this relationship.

**Electronic supplementary material:**

The online version of this article (doi:10.1186/s12931-017-0587-9) contains supplementary material, which is available to authorized users.

## Background

Chronic obstructive lung disease (COPD) is a well-known disease associated with a negative clinical outcome, including lung function decline and acute exacerbation (AE) [1]. The incidence and mortality of COPD have seen a global increase, and greater comprehension of the characteristics and management of this disease is needed [2–4].

The key pathophysiology of COPD is persistent and progressive airflow limitation [1]. However, airflow obstruction is reversible to some extent following the administration of a short-acting bronchodilator in many COPD patients. The prevalence of the positive bronchodilator reversibility (BDR) in COPD patients varies and has been reported as 15–50% [5, 6]. Studies have suggested that a positive BDR could be a phenotypic characteristic [7]. However, it remains unclear whether a positive response in the BDR test has an impact on the treatment outcome of COPD patients. One study reported that the response of patients response to pharmacological treatments cannot be prejudged by the acute response (reversibility) to short-acting bronchodilators [8]. A lack of an acute response to bronchodilators was not associated with a long-term response to maintenance bronchodilator treatment [9]. Several studies showed that COPD patients with a positive BDR were associated with a worse outcome such as increased risk of AE and re-hospitalization [10, 11]. On the other hand, other studies reported an association between BDR positivity and an improvement in the clinical course in COPD patients [12].

There is no established standard definition of relevant BDR [8], although different criteria for BDR positivity which have been used in a clinical context and in research, including the Global Initiative for Chronic Obstructive Lung Disease (GOLD) [1], American Thoracic Society (ATS) [13], American College of Chest Physicians (ACCP) [14], major criteria of the Spanish definition of asthma-COPD overlap syndrome [15], criteria compatible with ACOS in the Global Initiative for Asthma (GINA) [16], and European Respiratory Society (ERS) [17]. To the best of our knowledge, no study has yet compared the outcomes according to these BDR criteria.

The aim of this study was thus investigate the impact of positive BDR on the risk of severe AE according to different BDR criteria in COPD patients.

## Methods

We enrolled participants from four different prospective COPD cohort studies in South Korea: Seoul National University Hospital (SNUH) Airway Registry (NCT02527486), COPD in Dusty Area (CODA) Registry (KCT0000552), Korean COPD Subgroup Study (KOCOSS) (NCT02800499), and Korean Obstructive Lung Disease Cohort (KOLD). All COPD studies were registered with the exception of KOLD, which had been launched 12 years previously. The study design and methods were approved by the Institutional Review Board (IRB) of Seoul National University Hospital (IRB No. H-1507-030-686).

Participants who were over 40 years of age and who showed a post-bronchodilator forced expiratory volume in 1 s (FEV1)/forced vital capacity (FVC) < 0.7 were included. Those who were diagnosed with asthma, those not followed-up for at least 1 year, or those with a lack of information were excluded. Duplicate patients who were included in two or more cohorts were also excluded.

Various criteria for BDR positivity have been used both clinically and in research. Available definitions are distinguishable by the representative level of lung function (FEV1 [1, 14–17] or FVC [13], and whether to adopt an absolute or percentage change in that level. Several definitions use both absolute and percentage changes [1, 13, 15, 16] in pulmonary function to compensate for discordance in improvement from the baseline after bronchodilator application caused by the severity of COPD and thus provide a more comprehensive approach. The GOLD criteria for BDR positivity is an increase of 12% and 200 ml in post-bronchodilator FEV1 [1], the ATS criteria for BDR positivity is an increase of 12% and 200 ml in FEV1 or FVC [13], the ACCP requires an increase of 15% in FEV1 [14]. The major criteria of the Spanish definition of ACOS are an increase of 15% and 400 ml in FEV1 [15]. The criteria compatible with ACOS in GINA require an increase of 12% and 400 ml in FEV1 [16], while the ERS criteria are that post FEV1%- pre FEV1% ≥10% [17]. Finally, a criterion that was introduced by a study free of biases from sample size and sex was an 8% increase in FEV1 [12].

Baseline information of the study population, including demographic characteristics, smoking habits, and comorbidities was investigated in each cohort. Symptom scores from the COPD assessment test (CAT), St. George’s respiratory questionnaire (SGRQ), and the modified Medical Research Council (mMRC) dyspnea-scale were collected, as well as any severe AE event within 1 year prior to enrollment. Spirometry tests were performed using standardized equipment by qualified technicians following the ATS/ERS guidelines. Spirometry tests were repeated at least three times to achieve within- and between-maneuver acceptability criteria. After the initial spirometry testing (pre-dose spirometry), a dose of 100 μg of salbutamol was fully inhaled in one breath, and the breath was then held for 5 to 10 s before exhalation. Two separate doses (total dose 200 μg) were administered at approximately 30-s intervals. Three additional spirometry tests were performed between 10 and 15 min later for reversibility testing [18]. The medication possession ratios (MPRs) of the treatment drugs, including inhaled corticosteroid combined with long-acting beta agonists (ICS/LABA) and long-acting muscarinic antagonist (LAMA), were calculated as the total days of prescription days of each drug category divided by the total days of follow-up. All measurements for lung function were collected prospectively.

This study investigated the incidence of severe AE within 1 year of enrollment. All participants were asked to answer a questionnaire regarding the experience of AE of COPD since the previous visit at every follow-up visit. The definition of severe AE was any event that required an emergency room visit or hospital admission due to acute aggravation of COPD symptoms.

We compared those who experienced at least one severe AE event within 1 year to those without a severe AE event by using either a Mann-Whitney test or a Student’s *t*-test for continuous variables and a chi-squared test for categorical variables. Variables that showed a statistically significant difference between groups were included as adjustment covariates to investigate the effects of each set of BDR criteria on the incidence of severe AE in multivariable logistic models. Crude odds ratios (cORs), adjusted ORs (aORs), 95% confidence intervals (CIs), and the Akaike Information Criterion (AIC) were used to evaluate the models that included each set of BDR criteria as a principal variable. We carried out a sensitivity analysis for patients with a smoking history ≥10 pack-years (PY). The effects of treatment drugs (ICS/LABA and LAMA) and FEV1% on the relationship between each of the BDR criteria and severe AE were also explored. A propensity score for a positive BDR was also calculated by using various covariates and an analysis was conducted in the propensity score-matched participants. A *p*-value < 0.05 was regarded as statistically significant. SPSS 20.0 (IBM Corp., Armonk, NY, USA) and STATA 14.1 (StataCorp, TX, USA) were used for statistical analysis.

## Results

**Fig. 1:**
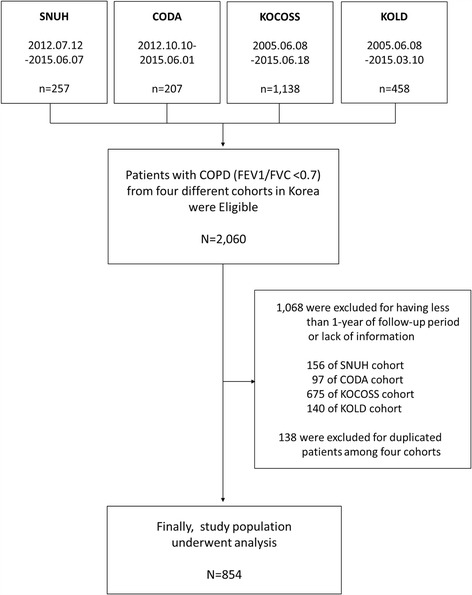
Flow chart showing the enrollment process for participants. *SNUH* Seoul National University Hospital Cohorts, *CODA* COPD in Dusty Area, *KOCOSS* Korean COPD Subgroup Study, *KOLD* Korean Obstructive Lung Disease Cohort

Among the patients in the four cohorts, patients with more than 1-year of follow-up data were selected. As shown in the flow chart for enrollment (Fig. 1), a total of 854 patients were included in this study.

The baseline characteristics of the patients are shown in Table 1. The mean CAT score was 15.4 (SD 7.9), and the mean SGRQ and mMRC scores were 33.1 (SD 17.3) and 1.61 (SD 1.01), respectively. About 10.9% of patients experienced severe AE at least 1 year prior to cohort enrollment. The initial mean value of FEV1 was 1.56 L (SD 0.55).

**Table 1:** Baseline characteristics of the participants

Characteristics	All participants (*n* = 854)
Age (mean, SD)	68.3 (7.6)
Male (N, %)	776 (90.9)
Cohorts registration (N, %)	
SNUH	101 (11.8)
CODA	110 (12.9)
KOCOSS	325 (38.1)
KOLD	318 (37.2)
BMI (mean, SD)	23.0 (3.4)
Weight (mean, SD)	61.7 (10.7)
Height (mean, SD)	163.6 (7.4)
Smoking habits	
None smoker	81/851 (9.5)
Ex-smoker	538/851 (63.2)
Current smoker	232/851 (27.3)
Pack-year (mean, SD)	43.3 (28.7)
Comorbidities (N, %)	
Diabetes mellitus	111/825 (13.5)
Heart disease	67/841 (8.0)
Cancer	24/519 (4.6)
Symptom scores	
CAT (mean, SD)	15.4 (7.9)
CAT ≥10 (N, %)	455/614 (74.1)
SGRQ (mean, SD)	33.1 (17.3)
SGRQ ≥25 (N, %)	258/418 (61.7)
mMRC (mean, SD)	1.61 (1.01)
mMRC ≥2 (N, %)	403/834 (48.3)
Severe acute exacerbation within 1-year before enrollment (N, %)	93 (10.9)
Pulmonary function test, mean(SD)	
Initial FVC post (L)/(%)	3.18 (0.82)/87.6 (18.6)
Initial FEV1 post (L)/(%)	1.56 (0.55)/60.9 (19.7)
Initial FEV1 ≥ 50% (N, %)	586 (68.6)
Initial FEV1/FVC ratio post, mean(SD)	49.2 (11.7)
BDR criteria, N (%)	
BDR >12% and 200 ml (FEV1) (GOLD)	167 (19.6)
BDR ≥12% and 200 ml (FEV1 or FVC) (ATS)	255 (29.9)
BDR ≥15% (FEV1) (ACCP)	187 (21.9)
BDR >8% (FEV1)	383 (44.9)
BDR ≥15% and 400 ml (FEV1) (Spanish ACOS)	30 (3.5)
BDR >12% and 400 ml (FEV1) (ACOS GINA)	30 (3.5)
Post FEV1% - pre FEV1 % ≥ 10% (ERS)	124 (14.5)

*SNUH* Seoul National University Hospital Airway Registry, *CODA* COPD in Dusty Area Registry, *KOCOSS* Korean COPD Subgroup Study, *KOLD* Korean Obstructive Lung Disease Cohort, *N* number, *SD* standard deviation, *NR* not recorded, *CAT* COPD assessment test, *SGRQ* St. George’s respiratory questionnaire, *mMRC* modified medical research council dyspnea scale, *FEV1* forced expiratory volume in one second, *FVC* forced vital capacity, *BDR* bronchodilator reversibility

Among the 854 patients, BDR positivity differed according to the criteria used for the response. The positive BDR rate ranged from 0.9 to 61.6% across the cohorts and according to BDR criteria. Among the criteria, the criterion of BDR > 8% FEV1 yielded a relatively high positive rate (33.6–61.6%) in every cohort compared to the other positive BDR criteria. The major criteria for ACOS in the Spanish guidelines (15% and 400 ml in FEV1) showed the lowest rate of BDR positivity among the criteria.

During the 1-year follow-up period, the MPR of ICS/LABA was 0.52 (SD 0.44), and the MPR of LAMA was 0.54 (SD 0.43). About 10% of patients experienced severe AE during the 1-year follow-up period, ranging from 5.5 to 12.0% in all cohorts. The highest rate of severe AE occurred in patients from the KOCOSS cohort. (Table 2)

**Table 2:** Treatment and outcomes of the participants

Characteristics	Total (*n* = 854)	SNUH (*n* = 101)	CODA (*n* = 110)	KOCOSS (*n* = 325)	KOLD (*n* = 318)
Treatment					
ICS/LABA use: yes (%)	544/836 (65.1)	53/91 (58.2)	20 (18.2)	201/317 (63.4)	270 (84.9)
ICS/LABA MPR, mean (SD)	0.52 (0.44)	0.44 (0.43)	0.13 (0.32)	0.49 (0.42)	0.70 (0.39)
LAMA use: yes (%)	564/830 (68.0)	61/91 (67.0)	39 (35.5)	250/311 (80.4)	214 (67.3)
LAMA MPR, mean (SD)	0.54 (0.43)	0.50 (0.43)	0.26 (0.40)	0.66 (0.39)	0.53 (0.43)
Severe acute exacerbation (%)	81/854 (9.4)	6/101 (5.9)	6/110 (5.5)	39/325 (12.0)	30/318 (9.4)

*ICS/LABA* inhaled corticosteroid/long-acting beta-agonist, *LAMA* long-acting muscarinic antagonist, *MPR* medication possession ratio, *SD* standard deviation

Several factors including body mass index (BMI), comorbidity of diabetes mellitus (DM), symptom scores, and the experience of severe AE before cohort enrollment were revealed to be significant in our analysis. Among the BDR criteria, GOLD (BDR >12% and 200 ml FEV1) and ATS (BDR ≥ 12% and 200 ml FEV1 or FVC) showed a difference in positive rates between the severe AE(+) group and the severe AE(-) group (Additional file 1: Table S1). Adjusted ORs were calculated by adjusting for BMI, symptom score of mMRC (≥2 vs. < 2), comorbidity of DM, initial FEV1% (≥50 vs. <50), ICS/LABA MPR, and severe AE before cohort enrollment. Use of the GOLD and ATS criteria was associated with a decreased risk of severe AE (aOR = 0.37, 95% CI = 0.15–0.91 for GOLD; aOR = 0.51; 95% CI = 0.28–0.96 for ATS). All seven BDR criteria increased the goodness of fit estimated by the AIC in each model, and the amounts of improvement were similar among the seven criteria. In the sensitivity analysis for patients with a smoking history ≥10 PY, BDR positivity from the GOLD criteria still predicted a significantly decreased risk of severe AE in COPD patients (aOR = 0.36, 95% CI = 0.14–0.95) (Table 3).

**Table 3:** Risk of severe acute exacerbation according to different BDR criteria

	cOR (95% CI)	*p*-value	aOR^a^ (95% CI)	*p*-value	AIC
All participants (*n* = 854)					
BDR >12% and 200 ml (FEV1) (GOLD)	0.30 (0.13–0.71)	0.01	0.37 (0.15–0.91)	0.03	471.470
BDR ≥12% and 200 ml (FEV1 or FVC) (ATS)	0.51 (0.28–0.90)	0.02	0.51 (0.28–0.96)	0.04	472.485
BDR ≥15% (FEV1) (ACCP)	0.73 (0.40–1.32)	0.29	0.53 (0.28–1.02)	0.06	473.416
BDR >8% (FEV1)	1.04 (0.66–1.64)	0.87	0.94 (0.57–1.55)	0.80	477.245
BDR ≥15% and 400 ml (FEV1) (Spanish ACOS)	0.32 (0.04–2.39)	0.27	0.51 (0.06–4.07)	0.53	476.726
BDR >12% and 400 ml (FEV1) (ACOS GINA)	0.32 (0.04–2.39)	0.27	0.51 (0.06–4.07)	0.53	476.726
Post FEV1% - pre FEV1 % ≥ 10% (ERS)	0.53 (0.24–1.18)	0.12	0.78 (0.33–1.84)	0.58	476.984
Participants with smoking history ≥ 10PY (a sensitivity analysis, *n* = 728)					
BDR >12% and 200 ml (FEV1) (GOLD)	0.29 (0.11–0.72)	0.01	0.36 (0.14–0.95)	0.04	399.119
BDR ≥12% and 200 ml (FEV1 or FVC) (ATS)	0.57 (0.31–1.05)	0.07	0.60 (0.31–1.15)	0.12	401.829
BDR ≥15% (FEV1) (ACCP)	0.70 (0.37–1.35)	0.29	0.56 (0.28–1.12)	0.10	401.406
BDR >8% (FEV1)	1.02 (0.61–1.69)	0.95	0.94 (0.54–1.64)	0.84	404.324
BDR ≥15% and 400 ml (FEV1) (Spanish ACOS)	0.35 (0.05–2.60)	0.30	0.61 (0.08–4.85)	0.64	404.022
BDR >12% and 400 ml (FEV1) (ACOS GINA)	0.35 (0.05–2.60)	0.30	0.61 (0.08–4.85)	0.64	404.022
Post FEV1% - pre FEV1 % ≥ 10% (ERS)	0.53 (0.22–1.26)	0.15	0.89 (0.35–2.24)	0.80	404.301

*BDR* bronchodilator reversibility, *PY* pack-year, *cOR* crude odds ratios, *aOR* adjusted ORs, *AIC* Akaike Information Criterion

^a^Adjusted by BMI, symptom score of mMRC ≥2 *vs* < 2, comorbidity of diabetes mellitus, initial FEV1% ≥50 *vs* < 50, medication possession ratio of inhaled corticosteroid/long-acting beta-agonist, and severe acute exacerbation within 1 year before enrollment

**Fig. 2:**
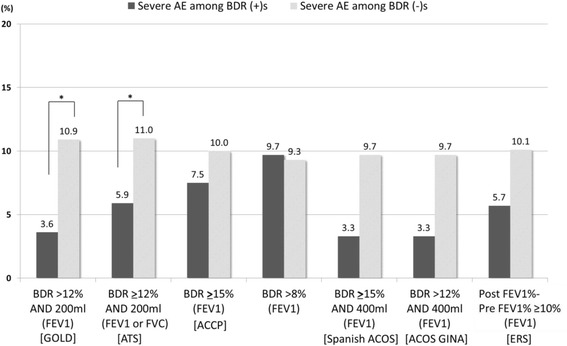
Proportion of patients with severe acute exacerbation according to BDR positivity. *with a statistical significance of *p*-value <0.05 *BDR* bronchodilator reversibility, *AE* acute exacerbation, *FEV1* forced expiratory volume in one second, *FVC* forced vital capacity

We compared the rate of severe AE between BDR positive and BDR negative patients during 1 year of follow up using different BDR criteria. Patients who showed BDR positivity experienced less severe AE than patients who showed BDR negativity when evaluated using the GOLD or ATS criteria (3.6% vs. 10.9%, *p* = 0.004 for GOLD, 5.9% vs. 11.0%, *p* = 0.02 for ATS) (Fig. 2).

**Fig. 3:**
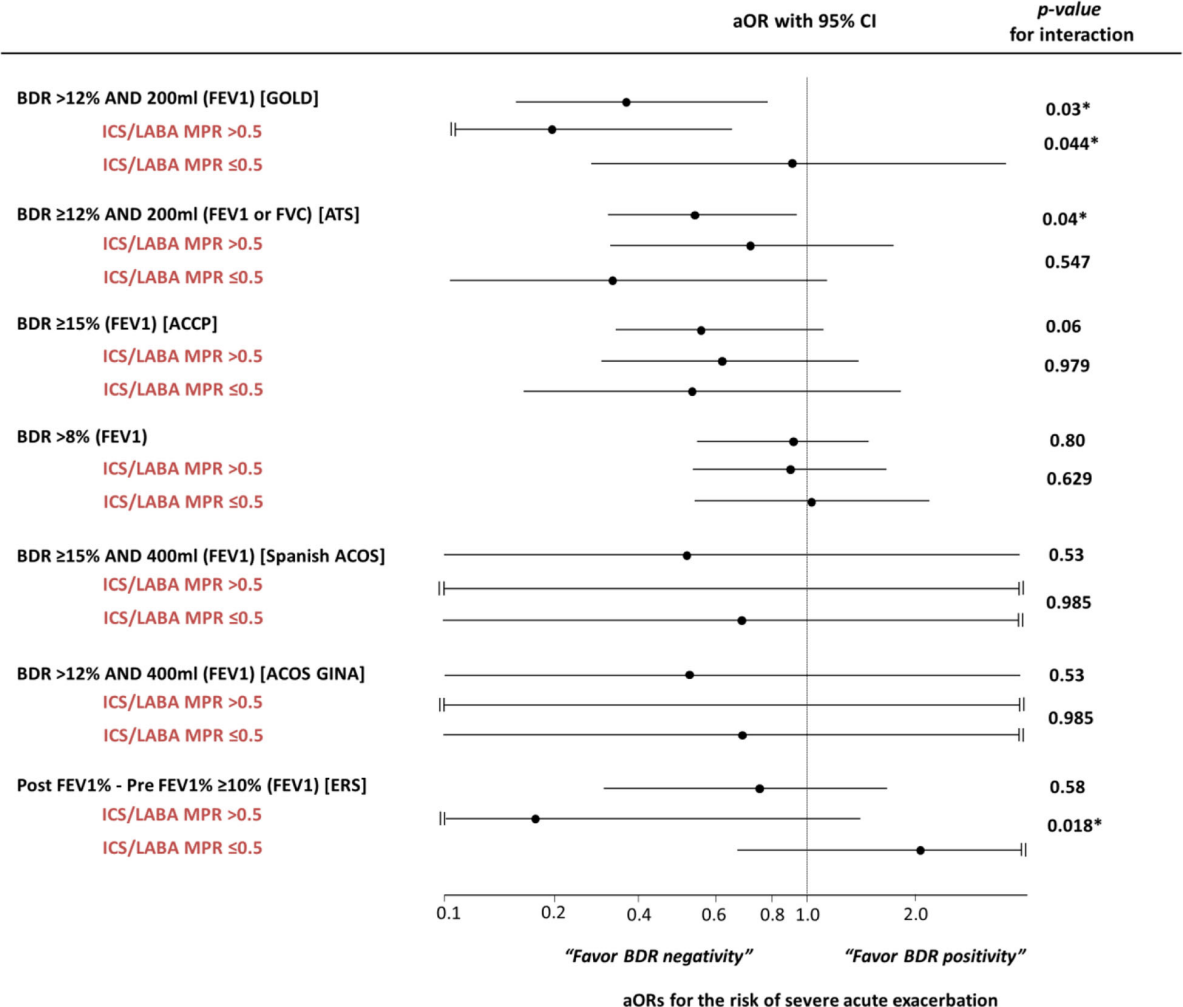
Risk of severe acute exacerbation according to BDR criteria including subgroup analysis according to ICS/LABA use. *aOR* adjusted odds ratio, *CI* confidence interval *BDR* bronchodilator reversibility, *MPR* medication possession ratio, *ICS/LABA* inhaled corticosteroid/long-acting beta-agonist, *LAMA* long-acting muscarinic antagonist, *FEV1* forced expiratory volume in one second, *FVC* forced vital capacity *with a statistical significance of *p*-value <0.05

When we calculated the risk of severe AE according to various BDR criteria stratified by ICS/LABA and LAMA MPR, there were significant interactions between ICS/LABA MPR and the GOLD or ERS criteria (post FEV1%-pre FEV1% ≥10%) (*p* = 0.044 and *p* = 0.018, respectively). If patients were treated with ICS/LABA for more than 6 months (MPR over 0.5), the rate of severe AE was reduced in cases of positive BDR according to GOLD or ERS criteria (Fig. 3 and Additional file 1: Table S2). Among patients with ≥10 PY, only BDR positivity according to the ERS criteria showed an effect modification through the use of ICS/LABA use (*p* = 0.021). There was no effect modification by LAMA occurred when modeling the effect of positive BDR on the risk of severe AE (Additional file 1: Table S2). In addition, no interaction was found between FEV1% (≥50% vs. <50%) or drug wash-out before enrollment (wash-out vs. no wash-out), and positive BDR on the risk of severe AE (data not shown). Even in the analysis f = of propensity score-matched participants, a positive BDR according to the GOLD criteria predicts severe AE. (aOR = 0.27, 95% CI = 0.10–0.75, *p* = 0.012) The balanced baseline characteristics between matched groups are presented in Additional file 1: Table S3.

## Discussion

To our knowledge, this is the first study to investigate the differences in treatment outcomes according to BDR criteria using prospective COPD cohorts. Our study showed that a positive BDR according to the GOLD criteria predicts a decreased risk of severe AE in COPD patients. The GOLD criteria showed a statistically significant relationship with the development of severe AE, and models using these criteria had the lowest AIC value. We did not find any significant association between BDR positivity and severe AE from other criteria (except ATS criteria in all participants). These results were consistent even in a sensitivity analysis which only included only patients with a smoking history of at least 10 PY.

The GOLD criteria require a >12% and 200 ml increase in FEV1 for a positive result. Although BDR criteria vary among various professional societies, and a standard definition has not been established [1, 13–17], reports suggest that a 12–15% increase in FEV1 compared to the baseline exceeds normal within-subject variability and response to placebo inhalation [19, 20]. When baseline FEV1 is low, a high improvement in the percentage can be possible with only a small improvement in the absolute volume. Because of this, use of an absolute volume increase of 200 ml has emerged as an alternative to using a percentage increase.

The usefulness of BDR positivity as a prognostic factor has been controversial [8, 11, 21]. BDR positivity favors a good treatment outcome in some studies, but demonstrates poor disease related outcomes in others. For example, Marin et al. reported a result similar to ours in that a positive BDR was significantly associated with a prolonged time to first hospitalization. However, theirs was a retrospective study [21]. Our results were somewhat different from PLATINO study, in which a positive BDR according to the ATS criteria and wheezing in the last 12 months showed a higher risk of hospitalization [11]. Self-reported wheezing might be a more severe symptom or a reflection of exacerbation, which could contribute to the worse outcome in these patients. This inconsistency might be due to the different study designs and ethnicity.

Our present study using prospective cohorts suggests that BDR positivity according to the GOLD criteria can predict a decreased risk of severe AE risk in univariate, multivariate, sensitivity and propensity score-matched analyses. Although the ATS criteria showed a similar result in all participants, statistical significance was not reached in a sensitivity analysis that excluded non-smokers and smokers with a history of less than 10 PY. Other criteria did not show a significant ability to predict severe AE. However, all aORs for positive BDR and severe AE were below 1.0 (range of aOR = 0.37–0.94). The mechanism of how BDR positivity in COPD patients leads to a decreased risk of severe AE has not been fully established, and there are several possible explanations for this. First, it could be the case that many asthma patients were misdiagnosed as positive BDR COPD patients. However, the sensitivity analysis excluded patients with a smoking history of less than 10 PY showed similar results, which suggests that asthma contamination alone is not an adequate explanation. Second, a positive BDR *per se* could predict a future positive response to drugs. Interestingly, in our study there was a significant interaction between ICS/LABA treatment and the effect of BDR on severe AE. A positive BDR according to the GOLD criteria predicted a decrease in the risk of severe AE only in patients who had an ICS/LABA MPR >0.5 (aOR for ICS/LABA MPR >0.5 = 0.18; 95% CI = 0.04–0.78; aOR for ICS/LABA MPR ≤0.5 = 0.95, 95% CI = 0.29–3.10; p for interaction = 0.044), although statistical significance was not reached in a sensitivity analysis that included only subjects with ≥10 PY. Our results suggest that a positive BDR could predict a response to ICS/LABA treatment. In support of this, it has been reported that increased reversibility of short-acting beta-2 agonists is associated with an increase in eosinophils and in exhaled nitric oxide (NO) [22]. This reversibility could be a phenotype of a good responder to inhaled corticosteroids [23–27]. We found there to be no effect modification by LAMA treatment for the effect of BDR on the incidence of severe AE in our study (all p for interactions >0.05).

This study has several key strengths. First, it is the first prospective study to compare clinical outcomes in COPD patients using various BDR positivity criteria. Second, this study was performed in a non-Western region and therefore represents the characteristics of COPD patients from non-Western countries. The results of this study could be helpful in future clinical trials or longitudinal studies. Third, a relatively large number of COPD patients from four different COPD cohorts were included in this analysis. The results of this study could therefore provide clinically significant information in a real-world setting. Last, we applied propensity score-matched analysis to strengthen the results.

This study also has several limitations. First, we did not investigate long-term outcomes, including mortality, owing to the limitations of the follow-up periods in these cohorts. Second, we did not have access to a large number of patients who had experienced severe AE. However, the number of patients with severe AE was sufficient to establish a multiple logistic regression model. Third, as a pooled analysis of four different cohorts, bias might have been present that rendered our sample unrepresentative of the total South Korean population. When we examined the patient demographic data of the four cohorts in this study, however, we found that they seemed to have similar baseline FEV1 and symptom scores. This allowed us to use a combined sample to represent COPD patients in South Korea. Fourth, small number of severe AE events in participants might have led to a lack of statistical power. Fifth, the main finding of this study might be comprise random statistical results due to an increase in type 1 errors from the multiple analyses; this was inevitable when determining if each of the BDR criteria was related to the risk of exacerbation. Sixth, 15 patients experienced multiple severe AE events during follow-up, which were not considered in the logistic models. Last, we only included participants who were followed-up for at least 1 year because the international guidance recommends that a study duration should be at least 1 year if the objective of the study is to investigate exacerbations [28]; this might form a selection bias by excluding many participants who were followed up for less than 1 year.

## Conclusions

The key pathophysiology of COPD is a persistent and progressive airflow limitation, that is reversible, to some extent, following the administration of a short-acting bronchodilator. Our results found that a positive BDR in the GOLD and ATS criteria could predict a decreased risk of severe AE in COPD patients. Even in the analysis for smokers, positivity in the GOLD criteria was still able to reflect the risk of severe AE of COPD. Longer ICS/LABA use provides a positive effect modification on this relationship. Other criteria for BDR positivity did not work for predicting the severe AE risk of COPD patients.

## Additional file

Additional file 1: Supplement data. (DOCX 44 kb)

## Abbreviations

AE: Acute exacerbation
AIC: Akaike information criterion
BDR: Bronchodilator reversibility
CAT: COPD assessment test
COPD: Chronic obstructive lung disease
FEV1: Forced expiratory volume in one second
FVC: Forced vital capacity
ICS: Inhaled corticosteroid
LABA: Long-acting beta agonists
LAMA: Long-acting muscarinic antagonist
mMRC: modified medical research council dyspnea scale
MPR: Medication possession ratio
SGRQ: St. George’s respiratory questionnaire
