# Supplementary material for: Which bronchodilator reversibility criteria can predict severe acute exacerbation in chronic obstructive pulmonary disease patients?
Source: Respir Res. 2017 May 30;18:107. doi: 10.1186/s12931-017-0587-9 (PMC5450062; doi:10.1186/s12931-017-0587-9)
Supplement: Additional file 1: — Supplement data. (DOCX 44 kb) [file 12931_2017_587_MOESM1_ESM.docx]

**Table S1.** Comparison of characteristics between severe AE (+) group vs. severe AE (-) group

| **Criteria** | **AE group (N=81)** | **No AE group (N=773)** | **p-value** |
| --- | --- | --- | --- |
| **Age, years, mean (SD)** | 69.3 (7.7) | 68.1 (7.6) | 0.61 |
| **Male, n (%)** | 70 (86.4) | 706 (91.3) | 0.28 |
| **BMI, mean (SD)** | 22.0 (3.7) | 23.1 (3.3) | 0.01 |
| **Weight, mean (SD)** | 57.3 (10.7) | 62.2 (10.6) | 0.0001 |
| **Height, mean (SD)** | 161.3 (8.1) | 163.9 (7.2) | 0.004 |
| **Smoking habits, n (%)** |  |  |  |
| None smoker | 11/79 (13.6) | 71/771 (9.2) | 0.06 |
| Former smoker | 57/79 (70.4) | 481/771 (62.4) |  |
| Current smoker | 13/79 (16.0) | 219/771 (28.4) |  |
| **Pack-year, mean (SD)** | 39.8 (27.6) | 43.7 (28.8) | 0.28 |
| **Comorbidities, n (%)** |  |  |  |
| Diabetes mellitus | 18/78 (23.1) | 93/746 (12.5) | 0.01 |
| Heart disease | 8/80 (10.0) | 59/760 (7.8) | 0.50 |
| Cancer | 3/42 (7.1) | 21/477 (4.4) | 0.42 |
| **Symptom scores** |  |  |  |
| CAT, mean (SD) | 20.4 (8.7) | 14.9 (7.6) | <.0001 |
| CAT ≥10, n (%) | 49/56 (87.5) | 406/558 (72.8) | 0.02 |
| SGRQ, mean (SD) | 44.3 (19.5) | 32.0 (16.7) | 0.001 |
| SGRQ ≥25, n (%) | 30/36 (83.3) | 228/382 (59.7) | 0.02 |
| mMRC, mean (SD) | 2.18 (0.96) | 1.55 (0.99) | <.0001 |
| mMRC ≥2, n (%) | 61/81 (75.3) | 342/753 (45.4) | <.0001 |
| **Severe acute exacerbation within 1-year before enrollment, n (%)** | 29 (35.8) | 67 (8.7) | <.0001 |
| **Pulmonary function test, post-bronchodilator, mean (SD)** |  |  |  |
| FVC, % of predicted | 80.9 (20.3) | 88.2 (18.3) | 0.001 |
| FEV1, % of predicted | 48.8 (19.5) | 62.1 (19.3) | <.0001 |
| FEV1 ≥50%, n (%) | 34 (42.0) | 552 (71.4) | <.0001 |
| FEV1/FVC | 42.5 (12.4) | 49.9 (11.4) | <.0001 |
| TLC, % of predicted | 115.5 (18.1) | 110.6 (24.6) | 0.36 |
| RV, % of predicted | 135.9 (45.6) | 130.8 (54.5) | 0.61 |
| RV/TLC | 45.5 (14.4) | 45.4 (13.0) | 0.96 |
| **BDR criteria, N (%)** |  |  |  |
| BDR >12% and 200ml (FEV1) (GOLD) | 6 (7.4) | 161 (20.8) | 0.004 |
| BDR ≥12% and 200ml (FEV1 or FVC) (ATS) | 15 (18.5) | 240 (31.1) | 0.02 |
| BDR ≥15% (FEV1) (ACCP) | 14 (17.3) | 173 (22.4) | 0.29 |
| BDR >8% (FEV1) | 37 (45.7) | 346 (44.8) | 0.87 |
| BDR ≥15% and 400ml (FEV1) (Spanish ACOS) | 1 (1.2) | 29 (3.8) | 0.35 |
| BDR >12% and 400ml (FEV1) (ACOS GINA) | 1 (1.2) | 29 (3.8) | 0.35 |
| Post FEV1% - pre FEV1 % ≥ 10% (ERS) | 7 (8.6) | 117 (15.1) | 0.11 |

N: Number, SD: Standard Deviation

AE: acute exacerbation, CAT: COPD assessment test, SGRQ: St. George’s respiratory questionnaire, mMRC: modified medical research council dyspnea scale, FEV1: forced expiratory volume in one second, FVC: forced vital capacity, BDR: bronchodilator reversibility

**Table S2.** Risk of severe acute exacerbation according to BDR criteria (subgroup analysis according to MPR of ICS/LABA and LAMA)

|  |  |  | **aOR* (95% CI)** | **p-value** | **P for interaction^‡^** | **P for interaction ^§^** |
| --- | --- | --- | --- | --- | --- | --- |
| **BDR >12% and 200ml (FEV1) (GOLD)** | |  |  |  |  |  |
|  | ICS/LABA MPR | > 0.5 | 0.18 (0.04-0.78) | 0.02 | 0.044 | 0.081 |
|  |  | ≤ 0.5 | 0.95 (0.29-3.10) | 0.94 |  |  |
|  | LAMA MPR | > 0.5 | 0.34 (0.08-1.52) | 0.16 | 0.422 |  |
|  |  | ≤ 0.5 | 0.35 (0.11-1.11) | 0.07 |  |  |
| **BDR ≥12% and 200ml (FEV1 or FVC) (ATS)** | |  |  |  |  |  |
|  | ICS/LABA MPR | > 0.5 | 0.66 (0.31-1.42) | 0.29 | 0.547 |  |
|  |  | ≤ 0.5 | 0.37 (0.12-1.17) | 0.09 |  |  |
|  | LAMA MPR | > 0.5 | 0.54 (0.22-1.35) | 0.19 | 0.898 |  |
|  |  | ≤ 0.5 | 0.49 (0.20-1.18) | 0.11 |  |  |
| **BDR ≥15% (FEV1) (ACCP)** |  |  |  |  |  |  |
|  | ICS/LABA MPR | > 0.5 | 0.59 (0.26-1.31) | 0.19 | 0.979 |  |
|  |  | ≤ 0.5 | 0.48 (0.15-1.58) | 0.23 |  |  |
|  | LAMA MPR | > 0.5 | 0.33 (0.11-0.95) | 0.04 | 0.342 |  |
|  |  | ≤ 0.5 | 0.69 (0.28-1.67) | 0.41 |  |  |
| **BDR >8% (FEV1)** |  |  |  |  |  |  |
|  | ICS/LABA MPR | > 0.5 | 0.93 (0.47-1.83) | 0.82 | 0.629 |  |
|  |  | ≤ 0.5 | 1.02 (0.45-2.28) | 0.97 |  |  |
|  | LAMA MPR | > 0.5 | 0.80 (0.38-1.69) | 0.55 | 0.463 |  |
|  |  | ≤ 0.5 | 1.15 (0.55-2.40) | 0.72 |  |  |
| **BDR ≥15% and 400ml (FEV1) (Spanish ACOS)** | |  |  |  |  |  |
|  | ICS/LABA MPR | > 0.5 | NC^†^ | 0.98 | NC^$^ |  |
|  |  | ≤ 0.5 | 1.44 (0.13-16.47) | 0.77 |  |  |
|  | LAMA MPR | > 0.5 | NC^†^ | 0.98 | 0.993 |  |
|  |  | ≤ 0.5 | 0.66 (0.07-5.97) | 0.71 |  |  |
| **BDR >12% AND 400ml (FEV1) (ACOS GINA)** | |  |  |  |  |  |
|  | ICS/LABA MPR | > 0.5 | NC^†^ | 0.98 | NC^$^ |  |
|  |  | ≤ 0.5 | 1.44 (0.13-16.47) | 0.77 |  |  |
|  | LAMA MPR | > 0.5 | NC^†^ |  | 0.993 |  |
|  |  | ≤ 0.5 | 0.66 (0.07-5.97) | 0.71 |  |  |
| **Post FEV1% - pre FEV1 % ≥ 10% (ERS)** | |  |  |  |  |  |
|  | ICS/LABA MPR | > 0.5 | 0.18 (0.02-1.36) | 0.10 | 0.018 | 0.021 |
|  |  | ≤ 0.5 | 2.11 (0.73-6.09) | 0.17 |  |  |
|  | LAMA MPR | > 0.5 | 1.10 (0.29-4.20) | 0.89 | 0.757 |  |
|  |  | ≤ 0.5 | 0.54 (0.16-1.76) | 0.30 |  |  |

ICS/LABA: inhaled corticosteroid/long-acting beta-agonist; LAMA: long-acting muscarinic antagonist; MPR: medication possession ratio, BDR: bronchodilator reversibility, aOR: adjusted odds ratios

* Adjusted by body mass index, symptom score of mMRC ≥2 *vs* < 2, comorbidity of diabetes mellitus, initial FEV1% ≥50 *vs* < 50, and severe acute exacerbation within 1 year before enrollment

† Not calculable

‡. Among all participants

§. Among participants with smoking history ≥ 10PY

**Table S3. The baseline clinical characteristics according to BDR positivity (GOLD definition) in propensity score matched participants.**

| **Characteristics** | **BDR (+)** | **BDR(-)** | **P-value** |
| --- | --- | --- | --- |
| **n** | 161 | 161 |  |
| **Age, years, mean (SD)** | 66.3 (7.7) | 67.0 (7.7) | 0.483 |
| **Male, n (%)** | 154 (95.7%) | 156 (96.9%) | 0.556 |
| **BMI, mean (SD)** | 23.9 (3.4) | 23.5 (3.4) | 0.346 |
| **Smoking habits, n (%)** |  |  | 0.778 |
| None smoker | 3 (1.9%) | 2 (1.2%) |  |
| Ex-smoker | 105 (65.2%) | 101 (62.7%) |  |
| Current smoker | 53 (32.9%) | 58 (36.0%) |  |
| **Pack-year, mean (SD)** | 43.7 (27.7) | 44.7 (25.1) | 0.725 |
| **Comorbidities, n (%)** |  |  |  |
| Diabetes mellitus | 14/159 (8.8%) | 7/159 (4.4%) | 0.287 |
| Heart disease | 7/159 (4.4%) | 4/159 (2.5%) | 0.655 |
| Cancer | 1/129 (0.8%) | 2/132 (6.3%) | 0.780 |
| **Symptom scores** |  |  |  |
| CAT ≥10, n (%) | 60/81 (74.1) | 54/77 (70.1%) | 0.777 |
| SGRQ ≥25, n (%) | 61/116 (52.6%) | 75/120 (62.5%) | 0.269 |
| mMRC ≥2, n (%) | 62/159 (39.0%) | 83/160 (51.9%) | 0.059 |
| **Severe AE within 1-year before enrollment, n (%)** | 14 (8.7%) | 18 (11.2%) | 0.456 |
| **Pulmonary function test, post-bronchodilator, mean (SD)** |  |  |  |
| FVC, L | 3.55 (0.73)) | 3.56 (0.77) | 0.850 |
| FEV1, L | 1.74 (0.45) | 1.72 (0.56) | 0.669 |
| FEV1/FVC | 49.4 (9.7) | 48.3 (11.5) | 0.351 |
